# Barriers to accessing substance abuse treatment in Mexico: national comparative analysis by migration status

**DOI:** 10.1186/1747-597X-9-30

**Published:** 2014-07-30

**Authors:** Erick G Guerrero, Jorge Ameth Villatoro, Yinfei Kong, Clara Fleiz, William A Vega, Steffanie A Strathdee, Maria Elena Medina-Mora

**Affiliations:** 1 School of Social Work, University of Southern California, 655 West 34th Street, Los Angeles, CA 90089, USA; 2 Instituto Nacional de Psiquiatría Ramón de la Fuente Muñiz, Calz. México Xochimilco 101, Col. San Lorenzo Huipulco, México D.F. C.P. 14370; 3 School of Medicine, University of California, San Diego, CA, USA

**Keywords:** Barriers to treatment, Migrant status, Gender, Mexico, Drug dependence

## Abstract

**Background:**

We examined Mexican migrants’ perceived barriers to entering substance abuse treatment and potential differences by gender.

**Methods:**

This study analyzed a subset of household data collected in Mexico in 2011 via the Encuesta Nacional de Adicciones (National Survey of Addictions). A sample of 1,143 individuals who reported using illicit drugs was analyzed using multivariate negative binomial models to determine direct and moderated relationships of gender, migrant status, and drug dependence with perceived barriers to accessing treatment.

**Results:**

Significant findings included disparities in drug dependence by migrant status. Compared with non-migrant men, women who have traveled to the United States was associated with fewer (1.3) barriers to access treatment. Fewer barriers to access care were associated with individuals residing in other regions of the country, compared to those living in Mexico City.

**Conclusions:**

Drug dependence, gender, migration status and regional location are factors associated with access to needed treatment. Implications for health care policy to develop treatment services infrastructure and for future research are discussed in the context of ongoing drug policy reform in Mexico.

## Background

Recent statistics have shown that the annual rate of illicit drug use in Mexico increased 87% between 2002 and 2011, from 0.8% to 1.5% [1]. In particular, women reported significant increases from 2008 to 2011 in the use of illicit drugs (marijuana and cocaine) [1]. Despite this growth in drug use, access to treatment for individuals reporting dependence has been low (19.8% for men and 8.8% for women), with significant variation among metropolitan and rural regions [1]. Drug trafficking, violence, and political turmoil have contributed to increased availability of drugs and illegal drug-related activities, placing communities with high migration and return migration levels, especially those in drug trafficking areas, at higher risk of drug use and potential need for treatment [2]. Mexico’s drug reform legislation enacted in 2008 defined threshold amounts of drugs for legal personal use and mandated treatment referral for those in possession of larger amounts [3]. In particular, recent findings have suggested that compared to Mexicans who have never visited the United States, risk of drug use is higher among highly mobile transnational men [4,5]. To respond to their potential need for treatment interventions, it is critical to examine the role of migration status, gender, and substance use in relation to access to treatment.

The high risk of drug use among transnational Mexicans, defined as individuals who have resided in both Mexico and the United States, may have negative effects on the health and well-being of residents in both countries [5-7]. Emerging evidence has suggested that Mexican migrants who come to the United States increase their drug use while in the United States and those who return to Mexico have higher rates of substance problems [4,5] that often go untreated, compared to the nonmigrant population [6,8,9]. Mexican migration to the United States has been associated with the transformation of substance use norms and pathology, with greater impact in border towns and metropolitan areas such as Tijuana, Ciudad Juarez, and Monterrey [10,11]. While residing in the United States, Mexican migrants are more likely to receive inadequate care and treatment compared to U.S.-born individuals of Mexican descent [12]. Because Mexico experienced one of the largest return migration from the United States from 2009 to 2012 [13] and women in particular face significant gender-related challenges to accessing treatment [14,15], it is imperative to evaluate perceived barriers to accessing substance abuse treatment (SAT) among migrants and potential gender differences in Mexico.

### Gender disparities in access to SAT

In the United States, gender disparities in access to SAT have been associated with limited availability of tailored services [14,15]. Although women are more likely to seek treatment for personal reasons (rather than due to a legal or employer mandate) [16], they often experience perceived (socially constructed) or structural barriers to entering treatment. Besides psychological barriers faced by drug users such as denial, minimization, and blaming, women are more likely than men to be affected by stigma and economic and family-related issues [17]. For instance, women with children need to secure child care to participate in outpatient SAT, and they may actively avoid residential programs due to fear of being required to relinquish their children as a condition of treatment [18-20]. Lack of adequate transportation is also a significant barrier to treatment access for both women and men [19,21]. In Mexico, stigma related to drug use has affected treatment engagement, and limited access to health insurance, poorly trained staff members, and scarce treatment options have reduced the likelihood that stigmatized populations (women and migrants) will access needed treatment [17,22]. In addition, despite the significant need for SAT, there are very few specialized programs (e.g., opiate treatment) operating in Mexico [11]. Understanding these barriers to treatment is critical to develop outreach interventions, engagement strategies, and tailored therapeutic processes that respond to perceived (e.g., denial, stigma) and concrete (e.g., no insurance, lack of providers) barriers to accessing needed care. Although drug policy reform in Mexico has mandated referral to treatment, there is a significant need for research on access to treatment, particularly among vulnerable groups with a high risk of drug use.

### Conceptual framework

Barriers to accessing substance abuse treatment are among the most significant documented challenges in health services research. Current models have suggested that individual characteristics and culturally embedded social role expectations and beliefs play a significant role in seeking care [14,23].

#### Migrants’ substance use and barriers to treatment

Migrants are more likely to experience emotional and physical vulnerabilities related to separation from their social networks, potentially leading to increased substance abuse and sexual risk behaviors [24]. These behaviors among migrant men have been strongly correlated with the absence of traditional family living arrangements and community patterns [25]. Migration is associated with separation from families and communities of origin, high stress related to labor and housing conditions, and exposure to alcohol abuse and drug use environments. Use of drugs and disconnection from social networks and communities among migrants may lead to more perceived barriers to accessing SAT compared to nonmigrant Mexicans.

#### Hypothesis 1

Compared with nonmigrant Mexicans who report ever using illicit drugs, transnationals who have used illicit drugs would report more barriers to accessing SAT.

#### Hypothesis 2

Compared with individuals who report using illicit drugs and drug dependence, individuals reporting illicit drug use but not dependence would report fewer barriers to accessing SAT.

#### Access to SAT by migrant status and gender

In a transnational context, women may face different challenges than men that affect their help-seeking behavior and perceived barriers to SAT treatment. Sex role socialization theories provide guidance for understanding gender disparities in drug use and access to care [19,23,26]. Compared with men, women are more frequently rewarded for caretaking roles, internalized emotional expression, and interdependent relations with others, leading to more barriers to accessing treatment related to child care, economic factors, and stigma. Furthermore, compared with nonmigrant women, barriers to accessing treatment may be more prevalent among transnational women, who also face disconnection from social networks and communities. This relationship may be further accentuated for individuals with drug dependence issues, whose perception of psychological barriers and experience with structural barriers may be more significant than occasional illicit drug users.

In the contrary, compared to non-migrant Mexicans, traveling Mexicans may be characterized as a population with higher resources that may reduce barriers to access treatment; traveling women in particular may be more likely than non-migrant men to report lower barriers to access care, as they may have more resources, but also higher pressures to be the care takers of their family. We expected to find differences in perceived access barriers by gender and migration status.

#### Hypothesis 3

The relationship between migration status and barriers to accessing SAT would be moderated by gender: (a) transnational women would report more barriers to accessing SAT compared with Mexican men and (b) traveling Mexican women would report fewer barriers to accessing SAT compared with Mexican men.

## Methods

### Data collection and procedures

This study analyzed a subset of data collected during the Mexico’s Encuesta Nacional de Adicciones (ENA), or the National Survey of Addictions. The ENA was a nationally representative survey collected by Mexico’s Instituto Nacional de Psiquiatría (National Institute of Psychiatry) from households in Mexico in 2011. The data used in this study were obtained from rural (fewer than 2,500 residents), urban (2,500 to 99,999 residents), and metropolitan (more than 100,000 residents) areas. Exclusion criteria included localities in which more than half of the population reported a native (indigenous) language that was not Spanish (e.g., dialect), due to the inadequacy of using interpreters to explore sensitive issues of addiction. As with other nationally representative surveys in Mexico and based on differences in substance use and utilization of services by size of municipalities, the sample was stratified by rural, urban, and metropolitan areas.

### Analytic sample and sampling procedures

We analyzed a sample of 1,143 subjects who reported using illicit drugs in their lifetime. We focused on using illicit drugs and did not include alcohol because recently enacted drug reform has mandated treatment for individuals who are caught with specified threshold amounts of illicit drugs.

A probability, multiphase, and stratified sampling procedure was applied to all primary sampling units. These units, which represented municipalities within states, were drawn from census tracts or geostatistical areas defined in Mexico’s census data collected in 2010. Municipalities within states were randomly selected with equal representation of rural, urban, and metropolitan areas. In each municipality, six blocks were randomly selected and six households were randomly selected from each block. Due to poorly defined blocks in rural areas, a cluster of 50 households was randomly selected, followed by a random selection of 12 households.

Data were collected during face-to-face interviews in households using a laptop computer. The survey considered adults (18–65 years of age) and adolescents (12–17 years of age) in each household. The head of the household and an adolescent were interviewed. The adolescent sample was not included in the current study because adolescents’ perception of access to treatment is generally different than that of adults [27]. To achieve representativeness and follow the framework of a previous survey collected in 2008 for comparative purposes, efforts were made to estimate regional proportions. The national response rate across all regions was 73.3% and the average response per household was 1.29 individuals.

### Measures

#### Dependent variable

The dependent variable was number of barriers to substance abuse treatment. The survey included 15 dichotomous measures that represented barriers to treatment access; for example, my insurance does not cover such treatment; treatment would cost too much money; I can solve this issue on my own; I do not know where to go; the treatment center is far away; I cannot get an appointment; and I feel shame or fear to be hospitalized. These measures were created based on the literature on access to care (see Marsh et al. [21]). The following five categories of barriers were developed: (1) cost or insurance issues; (2) desire to solve drug issue personally; (3) access to treatment issues; (4) stigma; and (5) denial of personal drug problem. After preliminary analysis of each category as potential binary outcomes, we decided to rely on number of barriers because we found no statistically significant differences among these categories by migrant status (see Table 1) and using number of barriers as an outcome responded to our general question about access to care across sampled regions of Mexico.

**Table 1:** Individual characteristics for those who have used drugs by migrant status using 2011 data

**Variable**	**Nonmigrant Mexicans**	**Traveling Mexicans**	**Transnational Mexicans**
	**(*n*** **= 620)**	**(*n*** **= 159)**	**(*n*** **= 253)**
Individual Factors			
Female*	21.6	24.5	8.7
Age (*M*, *SD*)*	32.9 (10.9)	36.6 (11.1)	38.0 (10.8)
Married*	35.6	36.5	39.9
Less than High school*	85.0	58.0	92.9
Num of dependents (*M, SD*)*	3.8 (1.7)	3.5 (1.7)	3.7 (1.9)
Public insurance	29	15.1	31.6
Private insurance*	1.6	11.9	2.4
Drug dependence*	13.2	11.9	16.2
Depression scale*	1.8 (0.8)	1.6 (0.7)	1.7 (0.8)
*Barriers to access treatment*			
Cost or insurance	5.9	5.6	5.3
Solve it by their own	19.7	16.1	17.1
No access to the treatment	5.5	4.3	4.5
Stigma	7.4	6.5	6.3
Denied problem	53.2	52.9	55.3
Mean number of barriers	1.9	1.7	1.8
*Region*			
Northcentral	14.8	32.1	24.9
Northwest*	14.2	23.9	15.0
Northeast*	10.2	14.5	8.7
East	17.1	11.9	21.7
Central	9.4	4.4	9.5
Mexico City*	13.9	7.5	4.0
Southcentral*	10.2	2.5	13.4
South*	10.3	3.1	2.8

*Note.* Figures represent percentage unless otherwise noted.

*Means or frequencies are different across groups at *p* < .05.

#### Explanatory variables

There were three main explanatory variables of interest. The first was migrant status, categorized as Mexicans with no history of immigration or travel to the United States (hereafter referred to as nonmigrant Mexicans), Mexicans who had traveled to the United States at least once (referred to as traveling Mexicans), and Mexicans who had lived in the United States (referred to as transnationals). These three categories were created based on individual responses to quantitative and qualitative survey items. Respondents were asked if they had ever been to the United States (quantitative) and the reason for any visit to the United States (qualitative). These qualitative responses were coded based on visiting (e.g., as a tourist) versus living in the United States for an undetermined period of time.

The second variable of interest was gender, particularly as a moderator between migrant status and barriers to treatment. The third variable was current dependence on illicit drugs. This dichotomous variable was created based on three DSM-IV [28] diagnostic criteria measuring self-reported symptoms related to tolerance, failure to fulfill major responsibilities, and withdrawal during the previous 12 months.

We adjusted the analysis based on several key factors related to barriers to treatment, including whether respondents reported less education than high school and having insurance. We also accounted for geographic differences based on regions of Mexico: north central, northwest, northeast, west, central, south, and south central, using Mexico City as the reference category.

### Data analysis

The initial analysis relied on Stata (version 12) and the survey procedure (SVY) to conduct two analyses. In the first step, we compared individual demographic characteristics, barriers to accessing treatment, and drug dependence across the three population groups via analysis of variance and chi-square global tests. The second step of analysis also relied on the survey procedure to conduct multivariate negative binomial regression analyses using the NBREG command with a log link function [29]. Negative binomial regression with robust standard errors was used to analyze number of perceived barriers, a count measure with overdispersion, i.e., its variance was much greater than its mean [30]. Although zero-inflated specification may be required for outcomes with a high number of zeros, our count measure did not need that approach when using the survey procedure. Compared to Poisson regression, which is generally used to model count data and has the same mean structure, negative binomial analysis is more efficient at modeling overdispersed outcomes using the extra parameter of exposure to an event [30,31].

In the second step, we examined two interaction effects: the relationships among (1) gender, drug dependence, and access barriers and (2) migration status, gender, and access barriers. The parameters presented in the negative binomial regression were expressed as incidence rate ratios (IRRs). IRRs can be interpreted as the estimated rate ratio for a 1-unit increase in the independent variable, given the other variables are held constant in the model. Not all variables analyzed in the first step were entered in the regression models in the second step due to collinearity with other independent variables. These variables were being married and having public or private insurance.

Finally, we relied on Stata MARGINS to produce estimated values (e.g., number of barriers) for the different subgroups. We graphed the values to display interactions based on gender, migration status, and drug dependence.

## Results

Table 1 shows the comparative analysis across different population groups for participants who had ever used drugs. Although differences in perceived access barriers were not statistically significant among the three groups, disparities in terms of demographic characteristics and health insurance were noted. The most significant differences involved drug dependence, private insurance and education. Transnationals reported the highest proportion of drug dependence (16.0%), compared to 13.2% and 12.0% for nonmigrant Mexicans and traveling Mexicans, respectively. Traveling Mexicans reported the highest rate of private health insurance and higher education than non-migrant Mexicans, as indicated in Table 1.

### Gender, migrant status, and access barriers

Findings did not support Hypothesis 1. This hypothesis posited that compared with nonmigrant Mexicans who reported using illicit drugs, transnationals and traveling Mexicans who had ever used illicit drugs would report more barriers to accessing SAT. See results in Table 2.

**Table 2:** Negative binomial regression on number of barriers to treatment in Mexico, 2011

**Variable**	**IRR**	***SE***	***p***	**95% C.I.**
Traveling to U.S.^a^	0.992	0.164	0.961	0.717, 1.373
Transnational^a^	1.015	0.143	0.917	0.770, 1.337
Drug dependence	1.937	0.249	0.000	1.504, 2.494
Traveling to U.S. x female^b^	0.311	0.115	0.002	0.150, 0.643
Transnational x female^b^	0.656	0.172	0.109	0.392, 1.099
Drug dependence x female^b^	1.084	0.610	0.886	0.358, 3.276
Female	1.014	0.173	0.935	0.725, 1.417
Age	0.993	0.005	0.875	0.983, 1.003
Less than High school	1.085	0.155	0.986	0.818, 1.437
*Region*^*c*^				
North central	1.657	0.381	0.028	1.055, 2.602
Northwest	1.618	0.383	0.043	1.016, 2.576
Northeast	2.522	0.557	0.000	1.634, 3.892
West	3.364	0.794	0.000	2.116, 5.348
Central	1.989	0.409	0.001	1.327, 2.980
South central	2.386	0.625	0.001	1.426, 3.992
South	2.008	0.667	0.036	1.046, 3.857

*Note.* CI, confidence interval; IRR, incidence rate ratio; SE, standard error. IRRs can be interpreted as the estimated rate ratio for a 1-unit increase in the independent variable, given the other variables are held constant in the model. For example, compared to non-dependent, individuals reporting drug dependence are associated with an increased ratio for number of barriers of IRR = 1.937, while holding all other variables in the model constant. The corresponding p-value is less than 0.001.

^a^Mexicans who have not visited the United States was reference category.

^b^Interaction term.

^c^Mexico City was reference category.

*p < .05, **p < .01, ***p < .001.

Findings supported Hypothesis 2, which posited that compared with individuals reporting illicit drug use and drug dependence, individuals reporting illicit drug use but not dependence would report fewer barriers to accessing SAT. Drug dependence was associated with more barriers (IRR = 1.937, 95% CI = 1.504, 2.494) after adjusting for other variables.

**Figure 1:**
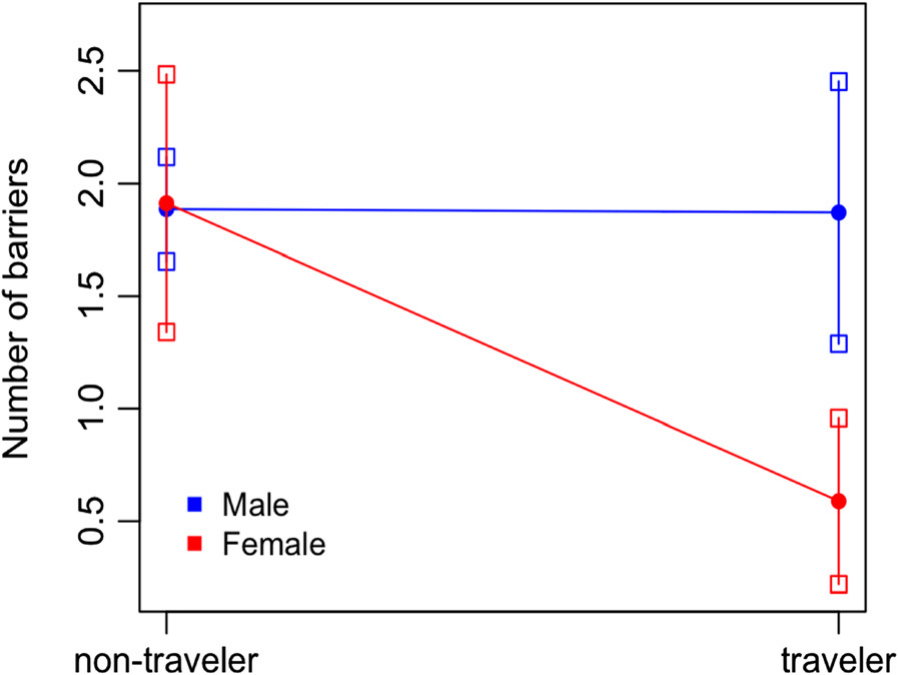
Predicted values of interaction term (gender and migrant status) on number of barriers.

Findings partially supported Hypothesis 3, which posited that the relationship between migration status and barriers to accessing SAT would be moderated by gender. The relationship between transnational Mexicans and barriers was not moderated by gender, providing no support for Hypothesis 3a. However, we found support for Hypothesis 3b, which posited that compared with nonmigrant Mexican men, women who visited the United States at least once reported fewer barriers to entering treatment (IRR = 0.311, 95% CI = 0.150, 0.643).The estimated values (e.g., number of barriers) for the different subgroups further showed that traveling Mexican women reported an average of 1.3 fewer barriers than nonmigrant men. Nonmigrant men reported 1.88 barriers, traveling men reported 1.87 barriers, nonmigrant women reported 1.91 barriers, and traveling women reported 0.59 barriers. The statistically significant interaction of gender and traveling Mexican status is presented in Figure 1.

Some control variables were directly related to barriers to accessing treatment. All regions in Mexico had higher incidences of reported barriers to entering treatment (*p* < .05) compared with Mexico City, the most populous metropolitan region with the largest health care infrastructure in the country.

## Discussion

Our analysis identified disparities in drug dependence by migrant status and highlighted the role of these two factors and gender in perceived barriers to access treatment. Transnationals reported the highest proportion of drug dependence (16%), compared with Mexicans who had traveled to the United States (13%) and Mexicans who had never visited the United States (12%). However, only the interaction of gender and immigration status was associated with number of individual barriers to accessing treatment. In particular, women traveling to the United States faced fewer barriers than the average nonmigrant Mexican men surveyed in the ENA. It has been well established that Mexican migrants are more susceptible than nonmigrant Mexicans to engaging in substance use and other risky behaviors [6,8]. Although migrants have reported a higher likelihood of using drugs and facing challenges to accessing care [4], in this study transnational did not perceive as many barriers to treatment as nonmigrant Mexicans.

Gender played a significant role in perceived barriers to accessing treatment, but only when considering migration status. Although women reported more barriers than men to entering treatment in other research on Latinos in the United States [17,32], findings suggested that among adults who have used illicit drugs, nonmigrant Mexican men reported an average of 1.3 more barriers to accessing substance abuse treatment compared to Mexican women who had traveled to the United States. This preliminary finding contributes to an initial understanding of differences in perceived access to care when considering groups categorized by migration status.

Unlike other studies that categorized Mexicans as migrants or not [6,8,10], this study further stratified Mexicans who had traveled to the United States to acknowledge this important and sizeable population. Sociodemographic characteristics of these individuals that may include their experience of traveling to the United States may differentiate their perceived barriers to accessing treatment compared to nonmigrants.

This is a significant contribution in terms of describing the diversity of Mexican adults exposed to the United States. It is important to note that individuals who had traveled to the United States generally reported more education and much higher rates of private insurance coverage and marriage than the other two groups. Albeit conjectural, these three factors may (imperfectly) represent socioeconomic status, suggesting that traveling Mexicans have more resources than Mexican nonmigrants and that higher socioeconomic status may play a role in their perceptions of increased access to needed care.

Our finding that individuals who reported drug dependence perceived more barriers to accessing treatment is consistent with national and international studies [17,33]. This finding highlights opportunities for further research focused on identifying differences in access to care for adults dependent on different types of illicit drugs.

Finally, differences in perceived barriers across national regions also represent a significant finding that needs to be investigated further. Compared to adults in Mexico City, the most populous and resource-rich region in the country in terms of health services infrastructure, adults who have used illegal drugs in other regions of the country reported more barriers to care. Future research should examine differences in psychological, social, and structural barriers across regions of Mexico, focusing on areas reporting the highest rates of illicit drug use activity such as the northern border region [8,10].

### Limitations

The limitations and strengths of this study are both related to characteristics of the ENA dataset. The representation of Mexicans in the ENA national household survey and questions about access to treatment are important strengths of this study. However, the ENA survey data were limited in terms of information collected on income and migration experiences, including deportation. These factors could help us further examine the heterogeneity among Mexicans with different migration experiences, particularly exposure to life in the United States. Another shortcoming of these data was the limited amount of information on individual characteristics that may limit generalizability of the findings, such as history of drug use, age of migration to and from the United States, length of stay in the United States, and legal status in the United States among transnationals. Age is also an important factor in perceived barriers to access care. However, preliminary examination of the interaction between age and migration status did not reach statistical significance (*p* > .05). Another limitation of this study includes not considering type of illicit drug(s) used, or access to different drug treatment modalities (e.g., outpatient, inpatient) accounting for program characteristics (e.g., certified vs. not, and public vs. private, the latter which incurs a fee). Overall, findings should be interpreted with caution because they describe the characteristics and experiences of adults surveyed in a household sample, which may not reflect the overall composition of Mexicans in Mexico or transnationals living in the United States. Despite these limitations, this study was one of the few and most current examinations of disparities in perceived access to treatment among Mexicans by three types of migration status using national household survey data from Mexico.

## Conclusion

Mexicans face barriers to accessing substance abuse treatment based on drug dependence, gender, migration status, and region. Drug-dependent adults with the highest need for treatment perceived the most barriers to accessing care, traveling Mexicans, who may have more resources, reported the fewest barriers. Limited community resources and personal norms, namely health services infrastructure and personal stigma related to receiving treatment, may also play a role in perceiving more barriers to accessing care, particularly among adults residing in nonmetropolitan and resource-poor regions.

Findings have implications for drug policy reform in Mexico, which in 2008 specified legal amounts of substances for limited personal use and affords three allowances (i.e., strikes) to people who are apprehended with quantities of drugs above the stated thresholds before they are sent to jail or mandated substance abuse treatment [3]. Mexico’s treatment infrastructure includes more than 480 treatment centers [34]. Nonetheless, this relatively limited treatment infrastructure in Mexico may compound individuals’ denial or minimization of their drug abuse issues as noted in this study, making it challenging for people to seek care, particularly in northern regions with high rates of drug use [8-10]. Policy makers should focus on reducing these regional disparities by enhancing access to free drug treatment at certified treatment centers that are tailored to the regional trends in drug use.

## Consent

Written informed consent was obtained from the patient’s guardian/parent/next of kin for the publication of this report and any accompanying images.

## Competing interests

The authors declare that they have no competing interests.

## Authors’ contributions

EG was responsible for conceptualization, writing, and revision of the manuscript. JV and YK were responsible for the creation of statistical models, data analyses, writing, and revision of the manuscript. CF, WV, SS and MM were responsible for writing and revision of the manuscript. All authors read and approved the final manuscript.
